# Characteristics of Febrile Children Admitted to the ICU Following an Unscheduled ED Revisit Within 72 h, a Case–Control Study

**DOI:** 10.3389/fped.2020.00411

**Published:** 2020-08-07

**Authors:** Charng-Yen Chiang, Yu-Lun Chen, Yan-Ren Lin, Fu-Jen Cheng, Kuan-Han Wu, I-Min Chiu

**Affiliations:** ^1^Department of Emergency Medicine, Kaohsiung Chang Gung Memorial Hospital, Kaohsiung, Taiwan; ^2^Department of Emergency Medicine, Changhua Christian Hospital, Changhua, Taiwan; ^3^School of Medicine, Kaohsiung Medical University, Kaohsiung, Taiwan; ^4^School of Medicine, Chung Shan Medical University, Taichung, Taiwan; ^5^Department of Computer Science and Engineering, National Sun Yet-sen University, Kaohsiung, Taiwan

**Keywords:** fever, children, intensive care unit admission, emergency department, unscheduled revisit

## Abstract

**Objective:** The purpose of this article was to demonstrate related characteristics of intensive care unit (ICU) admission after an unscheduled revisit by febrile children visiting the emergency department (ED).

**Method:** We performed a retrospective study in a tertiary medical center from 2010 to 2016. Patients whose chief complaint was fever and who were admitted to the ICU following a 72-h return visit to the ED were included, and we selected patients who were discharged from the same emergency department for comparison.

**Results:** During the study period, 54 (0.03%) patients met the inclusion criteria, and 216 patients were selected for the matched control group. Regarding clinical variables on initial ED visit, visiting during the night shift (66.7 vs. 46.8%, *p* = 0.010), shorter length of 1st ED stay (2.5 ± 2.63 vs. 3.5 ± 3.44 h, *p* = 0.017), and higher shock index (SI) (1.6 ± 0.07 vs. 1.4 ± 0.02, *p* = 0.008) were associated with ICU admission following a return visit. On the return ED visit, we found that clinical variables such as elevated heart rate, SI, white blood cell count, and C-reactive protein level were all associated with ICU admission. Furthermore, elevated SI and pediatric age-adjusted (SIPA) values were observed in the study group in both the initial (42.2 vs. 20.1%, OR:2.3 (1.37–4.31), *p* = 0.002) and return ED visits (29.7 vs. 6.9%, OR: 4.6 (2.42–8.26), *p* < 0.001).

**Conclusion:** For children who visited the emergency department with a febrile complaint, elevated SIPA values on the initial ED visit were associated with ICU admission following an unscheduled ED revisit within 72 h.

## Introduction

Since the 1980s, an unscheduled 72-h emergency department revisit has been considered an unsatisfactory outcome and has been widely studied (1–4). According to data from the past 10 years, the rate of return visits in pediatric emergency departments has been between 2.7 and 8.7% (5–10). About 7.1–19.7% of patients required admission after these revisits (5, 8), while 11% required ICU admission due to disease progression (8). According to previous studies, unlike other ED visitors, patients admitted to the ICU following an unscheduled return visit were associated with higher mortality and morbidity and were considered as having received a poor quality of care (11).

In a recent multicenter study, fever was the most common complaint for children revisiting the emergency department (ED) within 3 days (12). Fever primarily presents as a symptom of viral infection but can also be a sign of occult bacterial infection in 1.5–2% ED patients (13). Early recognition of bacterial infections with proper treatment for hemodynamic management and the use of antimicrobials can reduce children's mortality rate (14). Furthermore, the development or continuation of a critical condition after a return ED visit may cause great frustration and disappointment to caregivers (15). Therefore, determining high-risk febrile children who may develop unexpected complications and then making appropriate decisions based on their disposition is very important. The purpose of this study was to demonstrate the characteristics of febrile children admitted to the ICU within 72 h after ED discharge.

## Method

### Study Setting and Participants

This retrospective case–control study was conducted from 1 January 2010 to 31 December 2016 in the ED of a tertiary medical center in Southern Taiwan. The hospital had ~30,000 annual PED visits. This study was approved by the institutional review board of the Chang Gung Medical Foundation (IRB number: 101-4490B).

During the study period, we included previously healthy patients with febrile complaints, aged < 18 years, who revisited the ED within 72 h of previous ED discharge and were subsequently admitted to the ICU from ED as the study group. Patients with an underlying disease, who received an operation within 30 days, or who were discharged against medical advice (AAD) on the initial ED visit were all excluded. Physiologic status, represented by the Pediatric Risk of Mortality Score (PRISM) (16), and outcomes including mortality, ventilator assistance, ICU length of stay, and hospital length of stay of included patients were collected. All of patients' and physicians' records and information were anonymized and de-identified prior to review and analysis.

### Matched Control Group Selection

**Figure 1:**
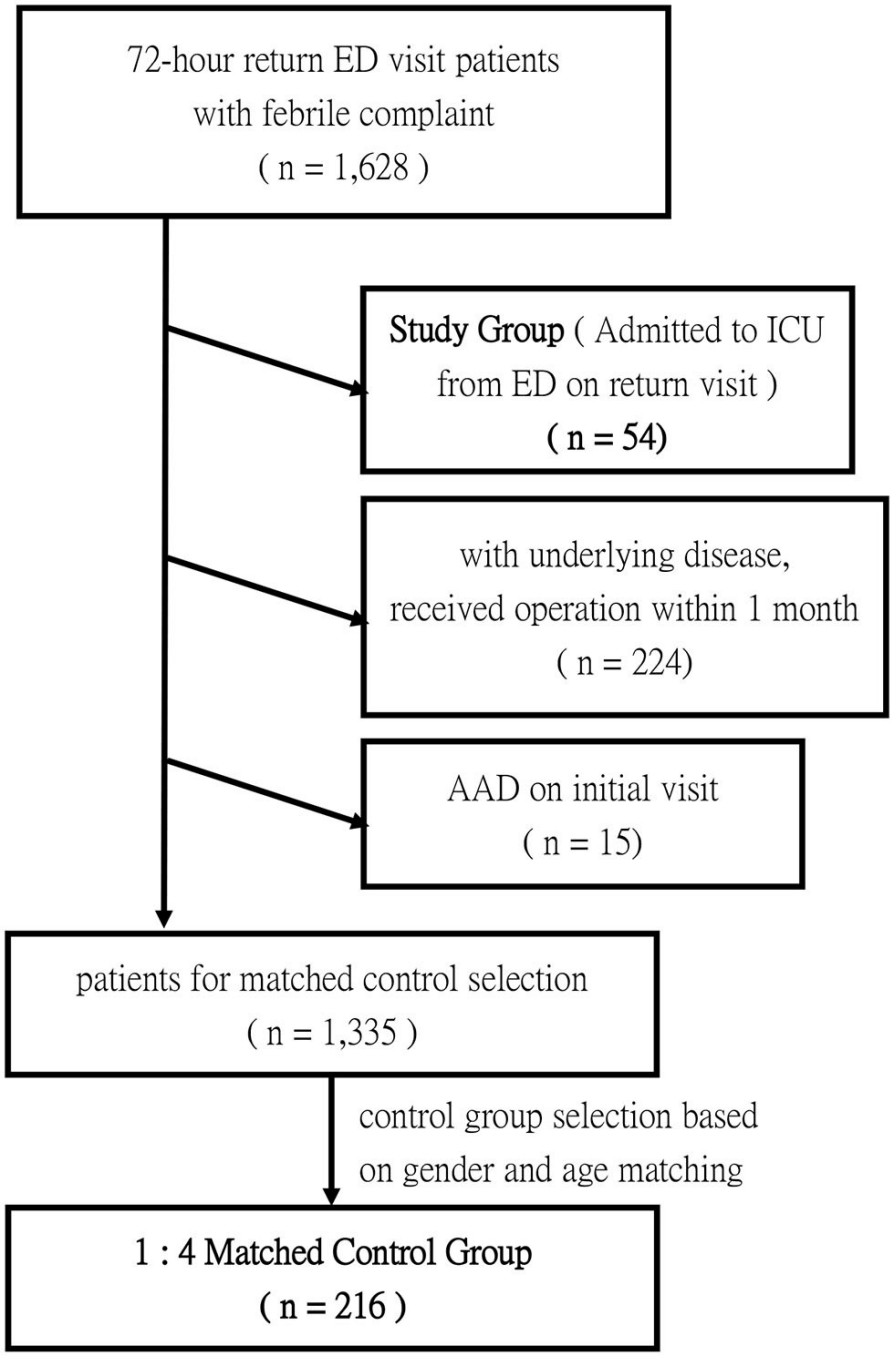
Flowchart illustrating inclusion criteria and matched control selection.

To investigate the characteristics and risk factors of patients with ICU admission on their return ED visit, we used a matched control group for comparison. To select the control group, patients with a febrile complaint who were discharged from the same ED and then revisited within 72 h were included for comparison. Of all the extracted patients, those with an underlying disease, who received an operation within 30 days, or who was discharged against medical advice on the visit were also excluded. Since study groups contain a limited number of patients with skewed distribution, we selected only an age- and gender-matched control group, instead of using all of the 72-h return visit patients. Stratified sampling of age matched cohorts was performed using the following five age brackets: under 3 months of age, from 3 to under 12 months of age, from 1 to under 3 years of age, from 3 to under 6 years of age, and 6–18 years of age. Patients who fulfilled the above criteria were picked randomly and assigned to the control group. The number of the control group was set as four times the study group. Figure 1 demonstrates the inclusion and matched control selection flowchart.

### Outcome Measurement and Analysis

After extracting the list of patients, we reviewed medical records such as electronic documented variables and ED visit logs. Patients' demographics, triage acuity, vital signs at triage, laboratory tests, and ED length of stay of both the initial ED visit and the return ED visit from the study group and the matched control group were recorded for risk factor analysis. Patients' reattendance window, defined as the time from the initial visit discharge to registration at the return visit, was also analyzed (17). Patient's triage level was categorized in terms of disease severity according to the Taiwan Triage and Acuity Scale computerized system in 2010, which was modified from the Canadian Triage and Acuity Scale (18). According to recommendation in Taiwan Society of Emergency Medicine, a higher acuity of triage, which defined as triage level above level II, indicate that a patient should be seen within 10 minutes from ED registration (18). Patients' vital signs at triage included body temperature (BT), heart rate (HR), and blood pressure (BP) and were measured at triage using an age-appropriate BP cuff size. We also considered time of ED visit, dividing patients' time of ED visit into day shift visits, which were defined as ED registration between 08:00 and 19:59 h, and night-shift visits, which were from 20:00 to 07:59 h.

In addition to such traditional vital signs as HR and BP, shock index (SI), defined as HR divided by systolic BP, was further calculated as a risk factor variable. We also applied SI, pediatric age-adjusted (SIPA) as a risk factor, which has been previously used as a poor indicator of severely injured children (19, 20). SIPA was defined as maximum HR divided by minimal systolic BP from an age-specific normal limit. Though previously used in the age range from 4 to 16 years, we have extended the range to cover 0–18 years based on the age-adjusted normal range suggested by Pediatric Advanced Life Support for comparison between the two groups (Table 1) (20, 21).

**Table 1:** Definition of normal vital signs and vital sign index (20, 21).

**Pediatric advanced life support vital signs by age group**			
	**HR, beats/min**	**SBP, mmHg**	**SI**
0–3 months	<160	<70	<2.1
3–12 months	<160	<80	<2.0
1–3 years	<150	<90	<1.6
3–6 years	<120	<100	<1.2
6–18 years	<110	<110	<1.0

Independent variables that may be associated with ICU admission following a PED revisit were analyzed with the chi-square test and nonparametric independent-sample test. Logistic regression was performed on the association of clinical variables with ICU admission on return visit after adjusting for other confounding factors. *P*-values of 0.05 were considered statistically significant. All statistical analyses were performed using SPSS Statistical Software (SPSS for MAC, version 22; SPSS).

## Results

During the study period, 210,694 pediatric patients were registered in our ED. Of those, 54 (0.03%) patients met the inclusion criteria and were included as the study group; the mean age was 1.9 ± 0.45 years, and 66.7% of them were male (Table 2). The median PRISM score during the 24 h of admission was 7 (3–13). Three (5.6%) of the patients expired during admission, and six patients (11.1%) required mechanical ventilator (MV) support. The average ICU length of stay was 4.9 ± 2.4 days, and the average hospital length of stay was 8.4 ± 1.07 days.

**Table 2:** Age and gender demographics and outcomes of ICU admission following an unscheduled ED revisit within 72 h.

**Variables**	**Studied group (*N* = 54)**
	**Mean** **±** ***SD*****/number (%)**
Male	36 (66.7)
Age (years)^*^	0.5 (0.2–1.6)
**Age Group**	
0–3 months old	17 (31.5%)
3–12 months old	19 (35.2%)
1–3 years old	9 (16.7%)
3–6 years old	4 (7.4%)
6–18 years old	5 (9.3%)
PRISM^*^	7 (3–13)
**Outcome**	
Mortality	3 (5.6)
Ventilator assistance	6 (11.1)
ICU length of stay	5.2 ± 2.11
Hospital length of stay	8.4 ± 1.07

*95% C.I., 95% confidence interval; PRISM, Pediatric Risk of Mortality Score; ICU, intensive care unit*.

* *Display as median (25–75th percentile)*.

The average length of the first ED stay was 2.5 ± 0.35 h, and the time interval to the second visit was 23.2 ± 2.34 h. Eighteen (33.3%) patients had their initial visit registered during the day shift, and 29 (53.7%) returned during the day shift.

After stratified sampling and selection, 216 patients were collected for the matched control group. Table 3 shows the comparison of clinical characteristics between the study group and the matched control group. Considering clinical variables from the initial ED visit, the triage vital signs of the initial visit demonstrated no statistical differences between the two groups. With regard to the return visit group, more patients visited during the night shift (66.7 vs. 46.8%, OR:2.3 (1.22–4.26), *p* = 0.010), and they had a shorter ED length of stay (2.5 ± 0.35 vs. 3.5 ± 0.21 h, *p* = 0.017). Considering the vital sign index, SI (1.6 ± 0.07 vs. 1.4 ± 0.02, *p* = 0.008) from the initial visit was also higher in the study group, and a more elevated SIPA was observed (42.2 vs. 20.1%, OR:2.3 (1.37–4.31), *p* = 0.002). However, none of the laboratory tests from the initial visit showed statistical differences. Logistic regression analysis on significant confounding factors from the initial ED visit showed that elevated SIPA values [aOR: 2.78 (1.153–6.716), *p* = 0.023] were independently associated with ICU admission following the return ED visit (Table 4).

**Table 3:** Comparison of clinical characteristics of the initial ED visit between early ED revisits with ICU admission and the control group.

	**Initial ED visit**		**MD/OR (95% CI)**	***p*-value**	**Return ED visit**		**MD/OR (95% CI)**	***p*-value**
	**Studied group**	**Matched control group**			**Studied group**	**Matched control group**		
Variables	Mean ± *SD*/*N* (%)	Mean ± *SD*/*N* (%)			Mean ± *SD*/*N* (%)	Mean ± *SD*/*N* (%)		
**Time of ED Visit**								
Day shift	18 (33.3)	115 (53.2%)	**2.3 (1.22–4.26)**	**0.010**	29 (53.7)	112 (52.1)	1.0 (0.76–1.28)	0.880
Night shift	36 (66.7)	101(46.8%)			25 (46.3)	103 (47.9)		
**ED length of stay (h)**	2.5 ± 2.63	3.5 ± 3.44	**1.1 (0.19–2.16)**	**0.017**	2.9 ± 3.13	3.6 ± 3.54	−0.7 (−2.33–1.02)	0.175
**Triage**								
Higher acuity	15 (27.8)	59 (27.3)	1.0 (0.54–1.48)	0.535	26 (48.1)	61 (28.2)	**1.8 (1.32–2.47)**	**0.005**
Lower acuity	39 (72.2)	157 (72.7)			28 (51.9)	155 (71.8)		
**Vital signs**	*N* = 42	*N* = 212			*N* = 53	*N* = 215		
BT (°C)	37.8 ± 1.21	37.3 ± 0.32	0.5 (−0.70–1.78)	0.788	37.9 ± 1.16	37.9 ± 1.30	0.2 (−1.12–1.38)	0.891
HR (/mins)	157 ± 30.4	152 ± 29.7	5.1 (−5.97–13.27)	0.613	150 ± 4.0	139 ± 2.0	**11.8 (2.88–21.12)**	**0.013**
SBP (mmHg)	100 ± 28.1	106 ± 19.6	−5.8 (−12.18–1.80)	0.069	107 ± 26.6	112 ± 18.7	−5.3 (−12.65–2.76)	0.092
DBP (mmHg)	63 ± 21.3	68 ± 19.6	−5.1 (−11.69–1.54)	0.100	67 ± 18.8	72 ± 15.6	−5.1 (−10.20–1.04)	0.079
**Shock index**	1.6 ± 0.07	1.4 ± 0.02	**0.2 (0.02–0.27)**	**0.030**	1.5 ± 0.46	1.3 ± 0.37	**0.2 (0.10–0.32)**	**0.015**
**Elevated SIPA**	19 (42.2%)	45 (20.1%)	**2.3 (1.37–4.31)**	**0.002**	16 (29.7)	15 (6.9)	**4.6 (2.42–8.26)**	**0.004**
**Laboratory test**	*N* = 28	*N* = 118			*N* = 53	*N* = 192		
WBC (k/μL)	8.4 ± 0.99	8.5 ± 0.31	−0.1 (−2.23–2.02)	0.906	17.5 ± 4.40	9.1 ± 1.34	**8.6 (3.14–13.92)**	**<0.001**
Neutrophil (%)	76 ± 2.3	69 ± 1.6	6.7 (−3.42–15.21)	0.546	79 ± 3.6	72 ± 2.8	7.3 (−4.12–19.06)	0.211
Hb (g/dL)	11.8 ± 0.37	12.1 ± 0.23	−0.4 (−1.96–1.14)	0.452	11.4 ± 0.37	11.8 ± 0.21	−0.4 (−1.22–1.02)	0.506
S ugar (mg/dl)	111 ± 15.2	105 ± 19.0	6.1 (−14.39–26.62)	0.556	118 ± 6.9	108 ± 16.3	10.4 (−2.32–24.08)	0.146
Na (mEq/L)	135 ± 2.4	137 ± 2.0	−2.2 (−8.42–4.15)	0.156	138 ± 3.1	137 ± 2.4	1.0 (−3.88–5.62)	0.197
K (mEq/L)	4.9 ± 1.52	4.3 ± 0.86	0.5 (−0.32–1.28)	0.264	4.6 ± 1.41	4.3 ± 0.90	0.3 (−0.26–0.78)	0.252
CRP (mg/L)	4.6 ± 2.73	12.4 ± 1.30	−7.8 (−16.91–2.35)	0.082	56.9 ± 11.59	18.4 ± 4.63	**39.0 (8.46–73.85)**	**<0.001**

*MD, mean difference; OR, odds ratios; SIPA=Shock Index, Pediatric Age-Adjusted*.

*Bold values are significant at α = 0.05*.

**Table 4:** Logistic regression analysis of clinical characteristics to early ED revisits with ICU admission after adjusting for age and gender.

	**aOR (95% CI)**	***p*-value**
Elevated SIPA	2.78 (1.153–6.716)	**0.023**
Initial visit during day shift	0.50 (0.232–1.068)	0.072
Length of initial ED stay	0.90 (0.760–1.052)	0.178

*Bold values are significant at α = 0.05*.

Regarding the return ED visit, patients admitted to the ICU after revisit were associated with a shorter reattendance window (23.2 ± 17.21 vs 30.0 ± 18.37 h, *p* = 0.015), higher triage acuity (48.1 vs. 28.2%, 1.8 (1.32–2.47), *p* = 0.005), elevated HR (150 ± 4.0 vs. 139 ± 2.0, *p* = 0.013), and both elevated SI (1.5 ± 0.46 vs. 1.3 ± 0.37, *p* = 0.005), and SIPA (29.7 vs. 6.9%, OR: 4.6 (2.42–8.26), *p* < 0.001). In laboratory tests, elevated values of WBC (17.5 ± 4.40 vs. 9.1 ± 1.34, *p* < 0.001) and CRP (56.9 ± 11.59 vs. 18.4 ± 4.63, *p* < 0.001) on the return visit were related to ICU admission.

## Discussion

In this retrospective cohort study, we focused on febrile children admitted to the ICU following an unscheduled ED return visit in an effort to determine associated risk factors from their initial visit. Among the predominant complaints for children visiting the ED, a return visit with fever was relatively common and is considered the result of “fever phobia,” which may require better medical education (22–24). Nevertheless, return ED visits with serious infection in febrile children, though rare, can be a critical issue and stressful on the physicians who manage such patients (25). In this study, we demonstrated an ICU admission rate of 0.5% following unplanned return visits, primarily of the male gender, which was similar to previous studies regarding the pediatric population (ranging from 0.4 to 0.7%) (8, 26). With a mean age of 1.9 ± 0.45 years for the included patients, we further separated them into five different age brackets for the selected comparison group since the likelihood of return visits varied with age intervals (12, 27).

Upon comparing the ICU admission and non-ICU admission groups, we found that time was one of the key points in determining risk factors. First, initial ED visits during the night shift were associated with return ICU admission in this study. A similar finding was also documented by Linden et al. and Goldman et al., where patients presenting during the night shift were more likely to return unscheduled (28, 29). Several studies have previously discussed the quality of care in the emergency department during the night shift. In such studies, the night-shift ED environment was reported to be associated with less patient evaluation, worse doctor–patient relationships, and worse resuscitation performance (30–33). These factors may contribute to a certain degree of ignorance with regard to detecting high-risk febrile patients. Second, patients with a shorter length of initial ED stay were associated with return ICU admission in this study. That more patients in the study group were visiting during the night shift for their initial visits may have also contributed to this finding. In the pediatric ED, certain high-risk febrile children may require more observation time to evaluate their clinical condition.

One study from 2015 had indicated that most children visiting the ED with vital signs of systemic inflammatory response syndrome (SIRS) were treated conservatively and discharged without critical illness or readmission (34). Said study also demonstrated that SIRS vital signs were actually very common among children, which accounts for more than 98% of ED visits in those presenting with a fever higher than 38.5°C. This conclusion can also be correlated with our study that vital signs from the initial visit showed no statistical differences between the ICU admission group and the control group. Nevertheless, shock index, a derivative from traditional vital signs, was found to be higher in the study group during the initial visit in our study. Compared to adults, children require more efficient assessment and treatment for critical illness. Previous studies have demonstrated that shock index was found to correlate with septic patients with worse clinical outcomes (35, 36). SI can indicate both stroke volume and systemic vascular resistance and has been encouraged as a triage tool for screening possibly septic patients (37). Our study also supported this by demonstrating that elevated SIPA at an initial ED visit was correlated with return ICU admission (Table 3). Since ICU admission following a return visit is seen as an indicator for rapid deterioration after ED discharge, an elevated SIPA should be considered before planning a patient's release. Regarding return ED visits, both SI values and traditional vital signs appeared different. Higher HR and also relatively lower SBP and DBP were observed in the ICU admission group, though without statistical significance. These effects also resulted in a higher acuity triage and may have affected physicians' decisions on patients' outlook. This obvious vital sign deterioration on return visit may contribute to shorter reattendance window of ICU admission patients.

Laboratory tests were also included for analysis but showed no statistical difference from their initial visit in this study. This finding may be limited since only 28 patients (51.9%) in the return ICU admission group and 118 (54.6%) in the comparison group received laboratory exams during their initial visit. Nevertheless, few studies in the past have demonstrated that laboratory testing could be used to distinguish between invasive infections in febrile children in the emergency department, and none of them showed beneficial accuracy (38–40). A large multicenter study conducted in 2017 further confirmed that no complete cell count could predict invasive bacterial infections in infants with high accuracy (41). On the other hand, almost every patient received a laboratory test on their return visit. Leukocytosis alone with elevated CRP levels was associated with ICU admission on the return visit. Along with worse vital signs at the return visit, laboratory tests also played an important role on patient outlook.

According to past studies, the reasons for return visits to the ED are multifactorial and usually reflect the natural progression of a disease rather than the quality of care received (8). However, ICU admission following an unscheduled revisit may not only put stress on the managing physician but also create a relationship of anxiety between the doctor and the family (11). Compared to the matched control group, this study suggested that elevated SIPA should be considered as an independent risk factor for ICU admission on return ED visit among febrile children. Higher SIPA levels should be further addressed in the future when making decisions regarding febrile children in the ED.

## Limitations

This study has several limitations. First, as a retrospective study with a limited database, some clinical characteristics were hard to collect and may fail to demonstrate significant difference during comparison. Nevertheless, most of the parameters demonstrated in this study were reasonable and relevant compared to previous studies. Second, admitting patients to the ICU may not always be a patient-centered decision. Mortality and morbidity after return ICU admission were rare, making these outcomes even harder to compare and analyze. Furthermore, some patients may have visited other hospitals after being discharged from the studied ED, but as the biggest pediatric referral center in the area, the likelihood of this problem should be low since ICU admission is the target inclusion criteria in this study.

## Data Availability Statement

Raw data were generated at Chang Gung Research Database (CGRD). Derived data supporting the findings of this study are available from the corresponding author on request.

## Ethics Statement

This study was approved by the Institutional Review Board of the Chang Gung Medical Foundation (IRB number: 101- 4490B).

## Author Contributions

C-YC analyzed and interpreted the patient data. Y-LC was a major contributor in writing the manuscript. Y-RL collected all data with initial pre-processing. F-JC was a contributor in writing the manuscript and the grammar check. K-HW constructed tables and statistical analysis. I-MC developed the concept for this article, performed statistical analysis, and supervised the work of this article. All authors read and approved the final manuscript.

## Conflict of Interest

The authors declare that the research was conducted in the absence of any commercial or financial relationships that could be construed as a potential conflict of interest.

## Abbreviations

ED: emergency department
ICU: intensive care unit
SI: shock index
SIPA: SI, pediatric age-adjusted
BT: body temperature
HR: heart rate
BP: blood pressure.
